# Mitochondrial Genome of *Grapsus albolineatus* and Insights into the Phylogeny of Brachyura

**DOI:** 10.3390/ani15050679

**Published:** 2025-02-26

**Authors:** Xue Zhang, Sheng Tang, Yaohui Chen, Qiuning Liu, Boping Tang

**Affiliations:** 1 College of Fisheries and Life Science, Shanghai Ocean University, Shanghai 201306, China; 2 Jiangsu Key Laboratory for Bioresources of Saline Soils, Jiangsu Synthetic Innovation Center for Coastal Bio-Agriculture, Jiangsu Provincial Key Laboratory of Coastal Wetland Bioresources and Environmental Protection, School of Wetlands, Yancheng Teachers University, Yancheng 224007, China

**Keywords:** gene arrangement, *Grapsus albolineatus*, mitogenomes, phylogenetic analysis, molecular evolution

## Abstract

Brachyura is among the most diverse groups of crustaceans, with over 7000 described species. Crab mitogenomes are important for understanding molecular evolution and phylogenetic relationships. *Grapsus albolineatus* exhibits specific rearrangements compared with the Pancrustacean ground pattern and other Brachyura species. The gene arrangement of *G. albolineatus* is similar to that of ancestral crustaceans, barring that of the translocated *trnH* gene. In phylogenetic analyses, the Bayesian inference estimation was observed to be superior to the maximum likelihood estimation when the nodal support values were compared. Considering the results of the gene rearrangement pattern and phylogenetic analysis, we speculate that *G. albolineatus* belongs to Grapsidae. Our comparative study indicated that mitogenomes are a useful phylogenetic tool at the subfamily level within Brachyura. The findings indicate that mitogenomes could be a useful tool for systematics in other Brachyuran species.

## Simple Summary

The diverse crustacean group Brachyura, which consists of over 7000 species, possesses important crab mitogenomes that are valuable for molecular evolution and phylogenetic studies. Among these species, *Grapsus albolineatus* stands out due to its unique rearrangements in comparison to the Pancrustacean ground pattern and other Brachyura species. Mitogenomes play a crucial role in subfamily-level phylogenetics within Brachyura and have the potential to aid in the systematic classification of other Brachyuran species.

## 1. Introduction

The mitochondria, an organelle of eukaryotic cells, has its own genome. It is commonly referred to as the mitochondrial genome. The mitochondrial genome, as a unique and easily accessible genetic marker, is characterised by a high mutation rate, high replication number, and maternal inheritance [1]. The metazoan mitochondrial genome is a double-stranded, circular, covalently closed molecule. Metazoans have a small mitochondrial genome, measuring approximately 16–20 kbp in length [2]. The mitochondrial genome comprises 13 protein-coding genes (PCGs), including seven nicotinamide adenine dinucleotide hydrogen (NADH) dehydrogenase subunits (NADH dehydrogenase subunit [ND] 1, ND2, ND3, ND4, ND5, ND6, and ND4l), cytochrome b (cytb), three cytochrome c oxidases (cytochrome C oxidase subunit [CO] I, COII, and COIII), and two adenosine triphosphate (ATP) synthase subunits (ATPase subunit [ATPase] 6 and ATPase8). Twenty-two transfer ribonucleic acid (RNA) genes (tRNAs), two ribosomal RNA genes (rRNAs), and a non-coding control region (also known as an adenine–thymine [AT]-rich region in invertebrates) regulate mitogenomic transcription and replication [3].

Nearly 7000 described species and 98 families make up the infraorder Brachyura [4]. The evolutionary history of the Brachyura can be viewed as an example of evolutionary radiation, where different directions of adaptive evolution (freshwater, marine, and intertidal) occurred due to different survival environments during the process of evolution. This ultimately resulted in the highly diverse group that we see today [5]. Correctly understanding the taxonomic status of species and constructing robust phylogenetic relationships are crucial for tracing the origin and evolution of the Brachyura. In the early stages, Guinot et al. [6] used the location of the male and female reproductive openings (gonopores) of crabs as a basis for classification of species in the Brachyura, and the Brachyura species were divided into three lineages, the Pototremata, the Heterotremata and the Thoracotremata. Under Guinot’s classification system, the Pototremata is divided into the Dromiacea and the Archaeobrachyura. The Heterotremata and the Thoracotremata are collectively referred to as Eubrachyura [7]. However, some other scholars did not use the theory of the Pototremata. Martin and Davis [5] identify the Dromiacea and the Eubrachyura as the two major taxa of the Brachyura. At the same time, Homolodromioidea in Archaeobrachyura was classified into Dromiacea, and Raninoidea and Cyclodorippoidea were placed under the newly created Rainoida in Eubrachyura. The results of the Tsang study showed that Homolodromioidea and Dromiacea have a sister-group relationship, providing support for Homolodromioidea to be classified into Dromiacea [8]. Other studies have shown that Homolodromioidea is more closely related to Raninoidea and Cyclodorippoidea, suggesting that Homolodromioidea should be moved from Dromiacea to the newly created Homoloida [9,10].

With the development of sequencing technology, particularly next-generation sequencing, the time and cost associated with sequencing have significantly decreased, making mitochondrial genome sequences easy to obtain [11]. Currently, mitochondrial genomic analysis is widely used in phylogenetic analysis, biogeography [12,13], population genetics [14], medicine, and ecology [15,16]. Tang et al.’s comparative study of the mitochondrial deoxyribonucleic acid (DNA) of *Helice wuana* and its relatives provides important evidence in terms of the origin, germline evolution, and the specific genetic structure formation of *H*. *wuana* [17]. The mitochondrial genome comprises 37 genes, theoretically possessing great rearrangement potential; however, based on the existing results, genetic rearrangements are infrequent. In conclusion, the same genetic rearrangement among different species is not likely due to convergent evolution but is more likely to predict a certain phylogenetic relationship among species. Therefore, analysing the genetic rearrangement is more suitable for understanding the superior phylogenetic relationship [18,19,20].

*Grapsus albolineatus* belongs to the genus *Grapsus* and is classified into Grapsoidea and Grapsidae. *G. albolineatus* is mainly found in Taiwan and Guangdong (including Hainan Island) in China, Japan, Hawaii, and Australia [21,22]. Herein, we sequenced the complete mitochondrial genome of *G. albolineatus* and compared it with other species of Brachyura. We inferred phylogenetic trees by analysing the amino acid and nucleotide sequences of 13 PCGs and analysed the genetic rearrangement patterns to understand the phylogenetic positions of *G. albolineatus* in Brachyura. At the end of the study, we downloaded other mitochondrial genomes of Brachyura crabs to obtain the phylogenetic status of Grapsoidea.

## 2. Materials and Methods

### 2.1. Sampling and DNA Extraction

We collected the mature crabs (*G. albolineatus*) from Xiamen, Fujian Province, China. Before DNA extraction, the specimen was stored in aerated tap water maintained at 21 ± 1 °C for 1 week. We extracted DNA from muscle tissue samples using an Aidlab Genomic DNA Extraction Kit (Aidlab, Beijing, China). The extracted DNA was stored at −20 °C until amplification.

### 2.2. PCR Amplification and Sequencing

Before amplifying the mitogenome of *G*. *albolineatus*, we designed a set of universal primers [17] using the Primer Premier 5.0 software. The primers were synthesised by the Beijing Sunbiotec Company, Beijing, China. We amplified DNA fragments using an Aidlab Extraction Kit per the manufacturer’s instructions. PCR was performed in a 50 μL reaction mixture containing 5 μL of 10× Taq plus Buffer (Mg^2+^), 4 μL of deoxynucleoside triphosphate, 2 μL of each of the primers, 2 μL of DNA, 34.5 μL of double-distilled water (ddH_2_O), and 0.5 μL of Taq DNA polymerase RED. The PCR conditions were as follows: 94 °C for 3 min, followed by 35 cycles of 30 s at 90 °C, annealing for 35 s at 49–58 °C (depending on the primer combination), elongation at 72 °C for 0.5–4 min (depending on the fragment length) and the final extension step at 72 °C for 10 min. We used agarose gel electrophoresis (1% *w*/*v*) to separate the PCR products and the Aidlab DNA Gel Extraction Kit for purification. Finally, we ligated the purified products into Tvector and sequenced them (Sangon Biotech, Shanghai, China).

### 2.3. Sequencing and Analysis

We annotated the sequence of *G. albolineatus* using the BLAST version 2.2.28 search function of the National Centre for Biotechnology Information (NCBI) (https://blast.ncbi.nlm.nih.gov/Blast.cgi (accessed on 12 March 2022)). The mitogenome of *G. albolineatus* was edited and assembled using the SeqMan software version 7 from the DNASTAR package (DNAStar Inc., Madison, WI, USA) [21]. It was visualised using the online tool OrganellarGenomeDRAW (OGDRAW) (https://chlorobox.mpimp-golm.mpg.de/OGDraw.html (accessed on 12 March 2022)), and the annotated sequence was represented on a graphical map [23]. The tRNAscan-SE webserver was used to predict the cloverleaf secondary structures of 22 tRNAs in *G. albolineatus* [24]. MEGA X was used to evaluate the relative synonymous codon usage value and nucleotide composition of the mitogenomes [25]. The nucleotide composition skewness was calculated using the following formulas [26]: AT skew = [A − T]/[A + T]; guanine–cytosine (GC) skew = [G − C]/[G + C].

### 2.4. Phylogenetic Analysis

The whole mitogenomes of 41 Brachyura species, along with their taxonomic status, were retrieved from the NCBI GeneBank. We used the mitogenome of *Alpueus distinguendus* as an outgroup taxon. The GeneBank ID is listed in Table 1. Subsequently, we used the Multiple Alignment using Fast Fourier Transform (MAFFT) selection for G-INS-i to align the nucleotide and amino acid sequences of 13 PCGs from these species with the invertebrate mitochondrial code [27]. We used Gblocks to identify and remove the unreliable parts of the MAFFT alignment results [28]. The 13 PCG sequences were combined using the Concatenate Sequence function of the PhyloSuite software version 1.2.3 [29]. In addition, we used Bayesian inference (BI) and maximum likelihood (ML) estimation. We selected the best molecular evolution model for the sequences for evolutionary inference using PartitionFinder2 [30]. Finally, we entered the Partitionfinder2 results into MrBayes v3.2 and IQ-TREE [31,32]. The MrBayes software version 3.2 ran for ten million generations with four chains and sampled every 100 generations with a 5000-generation burn-in step. The average standard deviation of the split frequency was <0.01, based on the convergence. The IQ-TREE software version 2 ran with 1000 bootstrapped replicates. The results were assessed using the Tracer v1.6 software. The effective sample size (ESS) value was >200 [33], which revealed that the simultaneous chains in the Markov chain Monte Carlo were convergent. The phylogenetic trees were visualised using the online tool, Interactive Tree Of Life [34].

## 3. Results and Discussion

### 3.1. Mitogenome Organisation and Nucleotide Composition

**Figure 1:**
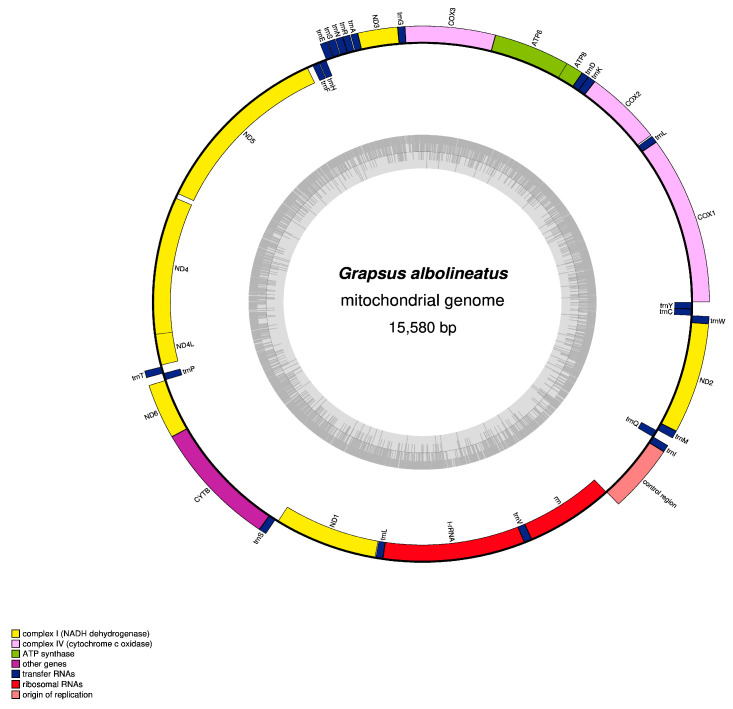
Circular map of the mitogenome of *Grapsus albolineatus*. Protein-coding and ribosomal genes are presented with standard abbreviations. Transfer RNA (tRNA) genes are shown by single-letter abbreviations, except for S1 = AGN, S2 = UCN, L1 = CUN, and L2 = UUR. The thick lines outside the circle indicate the heavy strand, whereas those inside the circle indicate the light strand.

The mitogenome of *G. albolineatus* is a closed circular molecule in structure with a length of 15,580 bp (Figure 1). Similar to that of other Brachyuran species, the mitogenome of *G. albolineatus* has 13 PCGs, 22 tRNAs, two rRNAs, and one AT-rich region. A total of 37 genes were observed, of which 14 (*trnH*, *trnF*, *nad5*, *nad4*, *nad4L*, *trnP*, *nad1*, *trnL1*, *rrnL*, *tranV*, *rrnS*, *trnQ*, *trnC*, and *trnY*) are transcribed on the light strand and 23 are transcribed on the heavy strand (Table 2). The nucleotide composition (A, 33.4%; T, 34%; C, 19.2%; and G, 11.8%) had a high AT bias. The AT-rich region constituted 67.4% of the total nucleotides. The calculated AT and GC skews were −0.01 and −0.26, respectively. Other listed Brachyuran species had negative GC skew values, and only nine species had a slightly positive AT skew value (Table 3).

### 3.2. Protein-Coding Genes

As presented in Table 2, the 13 PCGs ranged from 159 bp (*atp8*) to 1731 bp (*nad5*) in length. The PCG region in the mitogenome of *G. albolineatus* was 11,184 bp and composed of 13 genes (*nad1–6*, *nad4L*, *cox1–3*, *atp6*, *atp8*, and *cytb*). Twelve PCGs in *G. albolineatus* use the ATN codon as the start codon, except for the *atp8* gene, which has CTG as the start codon. Furthermore, ten PCGs contain a canonical stop codon (TCG (serine) or TAA (termination)), whereas the stop codons of the genes *cox1*, *cox*, and *cytb* contain only a single T nucleotide (Table 2).

**Figure 2:**
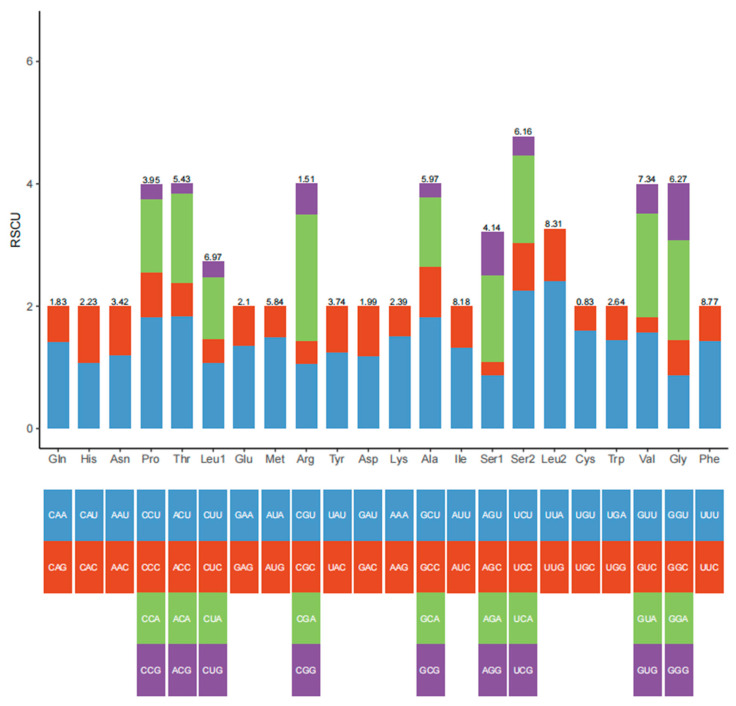
The relative synonymous codon usage (RSCU) values of the mitogenome of *Grapsus albolineatus*.

The codon usage of the PCGs is demonstrated in Table 4. The results revealed that the mitogenome of *G. albolineatus* has 3728 codons. The codon usage of *G. albolineatus* was skewed towards A or T. Other Brachyura species yielded similar results [35,36]. The relative synonymous codon usage values of *G*. *albolineatus* are indicated in Figure 2, which confirmed our results.

### 3.3. Transfer RNA and Ribosomal RNA Genes and Control Region

**Figure 3:**
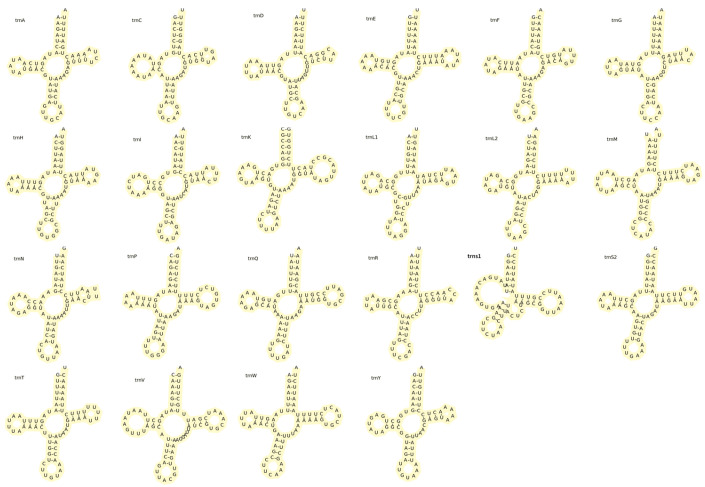
Putative secondary structure of the transfer RNA (tRNA) genes of the mitogenome of *Grapsus albolineatus*.

The two rRNAs (*rrnL* and *rrnS*) of *G. albolineatus* were 1328 bp and 827 bp in length, respectively. A *trnV* gene was observed between *rrnL* and *rrnS*; this arrangement is shared by other metazoans [37]. The mitogenome of *G. albolineatus* contains 22 tRNA genes (Figure 3, Table 2), each 64–71 nucleotides long, for a total length of 1480 bp. Furthermore, eight tRNAs (*trnH*, *trnF*, *trnP*, *trnL1*, *tranV*, *trnQ*, *trnC*, and *trnY*) were transcribed on the light strand. The cloverleaf structures of the tRNAs predicted by tRNAscan-SE are demonstrated in Figure 3. However, the *trns1* gene is an exception, since it has a bigger dihydroxyuridine arm and an extra arm. This finding is consistent with that of other Brachyuran species [38,39,40]. The control region between *rrnS* and *trnI* is 616 nucleotides long and contains the mitotic genome replication and transcription initiation site.

### 3.4. Gene Arrangement

**Figure 4:**
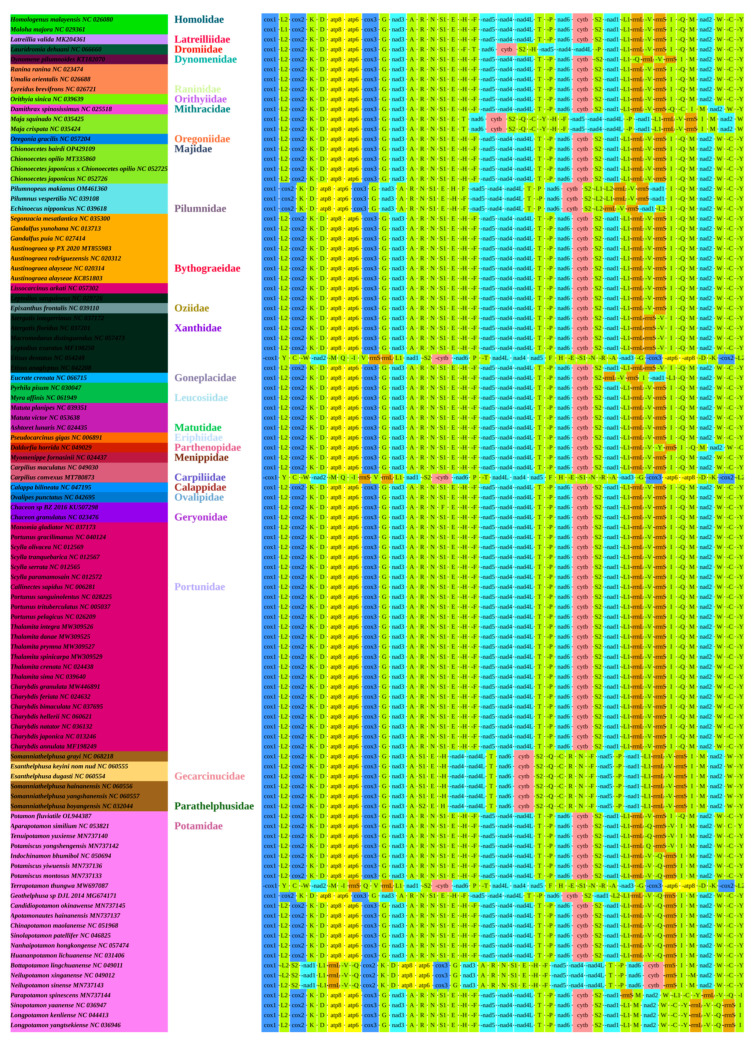
The gene order patterns of the Brachyuran species used in this study.

The arrangement of genes in the mitogenome is an effective tool to study phylogenetic relationships [41]. We compared the gene order of the whole mitogenome of *G. albolineatus* to the Pancrustacean ground pattern (*cox1*, *L2*, *cox2*, *K*, *D*, *atp8*, *atp6*, *cox3*, *G*, *nad3*, *A*, *R*, *N*, *S1*, *E*, *F*, *nad5*, *H*, *nad4*, *nad4L*, *T*, *P*, *nad6*, *cytb*, *S2*, *L1*, *rrnL*, *V*, *rrnS*, *CR*, *I*, *Q*, *M*, *nad2*, *W*, *C*, and *Y*) [42,43]. Except for the translocation of *trnH*, the gene sequence was observed to be identical (Figure 4). This phenomenon is not unique to *G. albolineatus*, as other Brachyuran species also exhibit similar tRNA rearrangements. We obtained eight gene order patterns from the selected Brachyuran species. The gene order of *G*. *albolineatus* is identical to that of the other seven families (Figure 4), suggesting a sister-group relationship. As demonstrated in Figure 4, the gene order of 13 PCGs and 2 rRNAs was unchanged, even though the tRNAs were translocated. The observed tRNA rearrangements, particularly the relocation of *trnH*, could be explained by several mechanisms. One hypothesis is tandem replication of partial mitogenomes followed by the loss of supernumerary genes [43,44,45]. However, this explanation may not fully account for the specific relocation of *trnH* without affecting other genes. An alternative hypothesis is tRNA remolding, a process in which tRNAs undergo structural and functional changes, potentially leading to their relocation within the mitogenome. tRNA remolding has been documented in metazoan mitochondrial genomes, where tRNAs can acquire new identities or functions through mutations in their anticodon loops or other structural modifications [46,47]. In the case of *G. albolineatus*, the relocation of *trnH* could be a result of such remolding events, which may have provided a selective advantage or been neutral in terms of evolutionary fitness. The gene order of *G. albolineatus* is consistent with that of other Brachyuran species, supporting its placement within the broader phylogenetic framework of Brachyura. However, the unique relocation of *trnH* raises questions about the evolutionary forces driving such changes. While the lack of recombination in mitochondrial genomes limits the mechanisms by which gene order can be rearranged, tRNA remolding offers a plausible explanation for the observed patterns. Further studies, including comparative analyses of tRNA sequences and structures across Brachyuran species, are needed to test this hypothesis and clarify the evolutionary history of *trnH* relocation. In contrast to *G. albolineatus*, the gene order of PCGs and tRNAs in Xenograpsidae species shows significant changes, consistent with previous studies [48,49]. This suggests that different evolutionary pressures or mechanisms may be at play in different Brachyuran lineages. On the other hand, the gene order of Varunidae species (*M. longipes*, *P. subquadrata*, and *E*. *sinensis*) is identical, supporting their classification within Grapsidae and Varunidae, as previously reported [50,51]. These findings highlight the diversity of mitogenome evolution within Brachyura and underscore the importance of considering both gene order and tRNA remolding in phylogenetic analyses.

### 3.5. Phylogenetic Analysis

**Figure 5:**
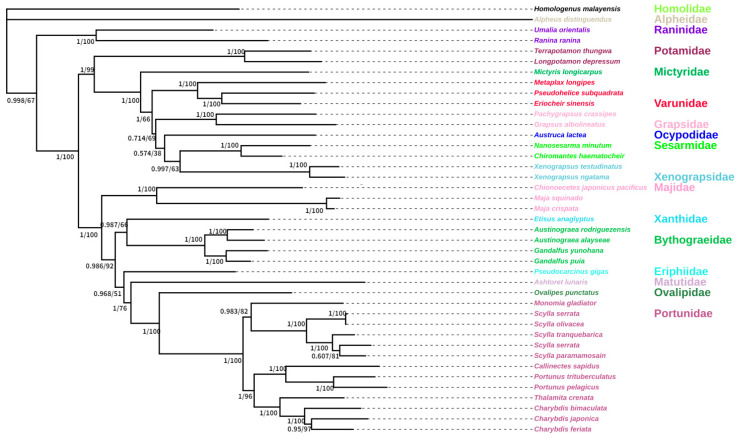
Phylogenetic tree inferred from the nucleotide sequences of 13 protein-coding genes (PCGs) of the mitogenome using Bayesian inference (BI) and maximum likelihood (ML) estimation. The Bayesian posterior probability (BPP) and bootstrap value (BP) of each node are shown as BPP/BP, with maxima of 1.00/100.

**Figure 6:**
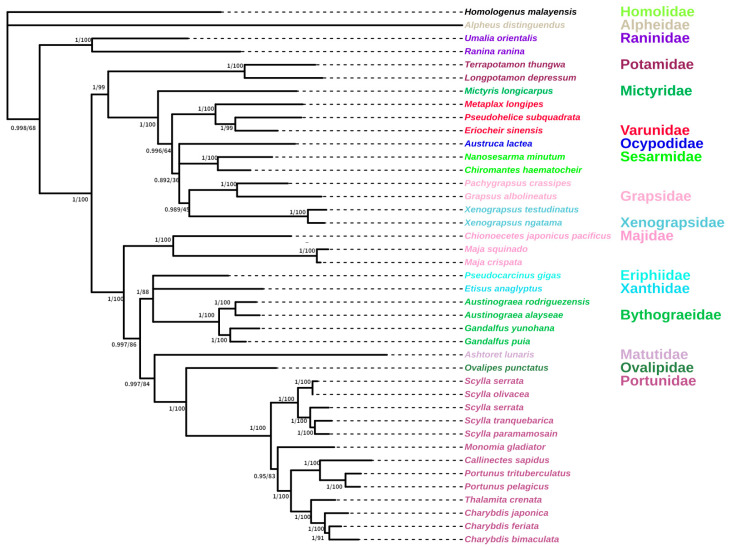
Phylogenetic tree inferred from the amino acid sequences of 13 protein-coding genes (PCGs) of the mitogenome using Bayesian inference (BI) and maximum likelihood (ML) estimation. The Bayesian posterior probability (BPP) and bootstrap value (BP) of each node are shown as BPP/BP, with maxima of 1.00/100.

We selected Brachyura species that were highly similar to *G. albolineatus* using the BLAST function of NCBI. We used both nucleotide and amino acid sequences of 13 PCGs in the 40 Brachyura species, listed in Table 1, to analyse the phylogeny of *G. albolineatus*. We constructed phylogenetic trees using BI and ML estimation with identical topologies (Figure 5 and Figure 6). *G. albolineatus* and *Pachygrapsus carssipes* were clustered in the same branch of the phylogenetic tree and had high nodal support values. Therefore, we hypothesised that they have a sister-group relationship, implying that *G. albolineatus* belongs to the Grapsidae family, which is consistent with the findings of a previous study [50].

The topologies of the phylogenetic trees were nearly identical to that observed in a previous study [10], particularly in the placement of *G. albolineatus* within Grapsidae. However, some discrepancies were observed, especially within the Grapsidae superfamily. For instance, the phylogenetic trees based on amino acid and nucleotide sequences showed different topologies in this group, and the nodal support values in certain regions were relatively low. The classification of Grapsidae has historically been complex due to morphological similarities and convergent evolution among species. Molecular data, while powerful, can sometimes yield conflicting results due to incomplete lineage sorting, hybridisation, or rapid diversification events [52]. Recent studies have highlighted the challenges in resolving Grapsidae phylogeny, particularly when using mitochondrial genomes alone [17,38,39]. Differences in evolutionary rates between nucleotide and amino acid sequences can lead to conflicting topologies. Amino acid sequences are generally more conserved, while nucleotide sequences may capture more recent evolutionary changes, leading to discrepancies in tree construction [51,53]. Mitochondrial gene order and tRNA rearrangements have been shown to play a significant role in Brachyuran phylogeny. Studies on *Helice latimera* and *Sesarmops sinensis* have demonstrated that gene rearrangements can provide additional phylogenetic signals, but they can also complicate tree reconstruction if not properly accounted for [39,54]. Recent advances in mitogenomic research have provided valuable insights into the phylogeny of Grapsidae and Brachyura. Studies on *Helice wuana* and *Clistocoeloma sinensis* have revealed extensive gene rearrangements and tRNA remolding events, which can influence phylogenetic reconstructions [17,43]. The ongoing development of DNA sequencing technologies and bioinformatics tools has revolutionised phylogenetic studies. High-throughput sequencing allows for the rapid generation of mitogenomic data, while advances in computational power enable the analysis of large datasets with complex evolutionary models [51,52]. These advancements will undoubtedly enhance our ability to resolve phylogenetic relationships within Brachyura and other taxa. With the emergence of long-read sequencing, previously unresolved or misclassified regions, such as those labelled as “non-coding” in invertebrates, have been increasingly clarified. This technological constraint may impact the completeness and accuracy of the assembled mitogenome, potentially overlooking critical genomic features [55]. Research on the higher phylogeny of Brachyura has highlighted the importance of integrating multiple data types, including nuclear and mitochondrial genomes, to resolve deep evolutionary relationships [56,57].

## 4. Conclusions

In this study, we analysed the whole mitogenome of *G. albolineatus.* Using gene arrangement patterns and phylogenetic analysis, we suggested that *G. albolineatus* belongs to the Grapsidae family. Meanwhile, using phylogenetic trees based on amino acid and nucleotide sequences, we discovered that the topologies in the Grapsoidea superfamily differed and the nodal support values in the area were low. We believe the classification of the Grapsoidea superfamily was not ideal. With the rapid advancement of the PCR technique, further research on the mitogenome will aid in more accurate classification of crabs.

## Figures and Tables

**Table 1:** List of the Brachyuran species used in this study.

Species	Family	Size (bp)	Accession No.
*Grapsus albolineatus*	Grapsidae	15,580	MF198247
*Chionoecetes japonicus pacificus*	Majidae	15,341	AB735678
*Scylla serrata*	Portunidae	15,721	HM590866
*Scylla paramamosain*	Portunidae	15,824	JX457150
*Austinograea alayseae*	Bythograeidae	15,611	KC581803
*Umalia orientalis*	Raninidae	15,466	KM365084
*Eriocheir sinensis*	Varunidae	16,350	KP064329
*Gandalfus puia*	Bythograeidae	15,548	KR002727
*Maja crispata*	Majidae	16,592	KY650651
*Maja squinado*	Majoidea	16,598	KY650652
*Xenograpsus ngatama*	Xenograpsidae	16,106	KY985236
*Ashtoret lunaris*	Matutidae	15,807	LK391941
*Mictyris longicarpus*	Mictyridae	15,548	LN611670
*Metaplax longipes*	Varunidae	16,424	MF198248
*Charybdis bimaculata*	Portunidae	15,712	MG787408
*Terrapotamon thungwa*	Potamidae	16,156	MW697078
*Portunus trituberculatus*	Portunidae	16,026	NC_005037
*Callinectes sapidus*	Portunidae	16,263	NC_006281
*Pseudocarcinus gigas*	Eriphiidae	15,515	NC_006891
*Scylla serrata*	Portunidae	15,775	NC_012565
*Scylla tranquebarica*	Portunidae	15,833	NC_012567
*Scylla olivacea*	Portunidae	15,723	NC_012569
*Charybdis japonica*	Portunidae	15,738	NC_013246
*Xenograpsus testudinatus*	Xenograpsidae	15,798	NC_013480
*Gandalfus yunohana*	Bythograeidae	15,567	NC_013713
*Alpheus distinguendus*	Alpheidae	15,700	NC_014883
*Austinograea rodriguezensis*	Bythograeidae	15,611	NC_020312
*Pachygrapsus crassipes*	Grapsidae	15,652	NC_021754
*Ranina ranina*	Raninidae	15,557	NC_023474
*Thalamita crenata*	Portunidae	15,787	NC_024438
*Charybdis feriata*	Portunidae	15,660	NC_024632
*Homologenus malayensis*	Homolidae	15,793	NC_026080
*Portunus pelagicus*	Portunidae	16,157	NC_026209
*Monomia gladiator*	Portunidae	15,878	NC_037173
*Nanosesarma minutum*	Sesarmidae	15,637	NC_040977
*Chiromantes haematocheir*	Sesarmidae	15,899	NC_042142
*Etisus anaglyptus*	Xanthidae	16,435	NC_042208
*Austruca lactea*	Ocypodidae	15,659	NC_042401
*Pseudohelice subquadrata*	Varunidae	16,898	NC_042685
*Ovalipes punctatus*	Ovalipidae	16,084	NC_042695
*Longpotamon depressum*	Potamidae	16,537	NC_057478

**Table 2:** Annotation of the complete mitochondrial genome of *Grapsus albolineatus*.

Gene	Direction	Location	Size (bp)	Intergenic Nucleotides	Start Codon	Stop Codon
*cox1*	F	1–1534	1534		ATG	T
trnL2	F	1535–1599	65			
*cox2*	F	1610–2297	688		ATG	T
trnK	F	2298–2366	69			
trnD	F	2367–2430	64			
*atp8*	F	2431–2589	159		GTG	TAA
*atp6*	F	2583–3257	675	−7	ATT	TAA
*cox3*	F	3257–4048	792	−1	ATG	TAA
trnG	F	4048–4110	63	−1		
*nad3*	F	4111–4461	351		ATC	TAA
trnA	F	4460–4523	64	−2		
trnR	F	4530–4593	64			
trnN	F	4595–4659	65			
trnS1	F	4664–4730	67			
trnE	F	4733–4800	68			
trnH	R	4804–4868	65			
trnF	R	4873–4937	65			
*nad5*	R	4990–6720	1731		ATG	TAA
*nad4*	R	6765–8102	1338		ATG	TAG
*nad4L*	R	8096–8398	303	−7	ATG	TAA
trnT	F	8413–8478	66			
trnP	R	8479–8545	67			
*nad6*	F	8548–9051	504		ATT	TAA
*cytb*	F	9051–10,185	1135	−1	ATG	T
trnS2	F	10,186–10,253	68			
*nad1*	R	10,281–11,246	966		ATT	TAA
trnL1	R	11,252–11,318	67			
rrnL	R	11,319–12,646	1328			
trnV	R	12,647–12,719	73			
rrnS	R	12,720–13,546	827			
CR	-	13,547–14,762	616			
trnI	F	14,164–14,229	67			
trnQ	R	14,227–14,297	71			
trnM	F	14,305–14,375	71			
*nad2*	F	14,376–15,386	1011		ATG	TAG
trnW	F	15,385–15,453	69	−2		
trnC	R	15,453–15,516	64	−1		
trnY	R	15,517–15,680	64			

**Table 3:** Nucleotide composition and skewness of the mitochondrial genome.

Species	Size (bp)	A (%)	T (%)	C (%)	G (%)	A + T (%)	A + T Skew	C + G Skew
*G. albolineatus*	15,580	33.4	34	20.5	12.1	67.4	−0.01	−0.26
*C. jpacificus*	15,341	34.6	37	17.2	11.1	71.6	−0.033	−0.215
*S. serrata*	15,721	33.4	35.8	19.5	11.3	69.2	−0.034	−0.266
*S. paramamosain*	15,824	34.9	38.2	16.8	10.2	73.1	−0.045	−0.247
*A. alayseae*	15,611	34.5	32.4	21.9	11.3	66.9	0.032	−0.321
*U. orientalis*	15,466	33.1	34.9	20.2	11.8	68	−0.027	−0.262
*E. sinensis*	16,350	35.3	36.4	17.6	10.7	71.7	−0.015	−0.245
*G. puia*	15,548	35.1	34.8	19.8	10.3	69.9	0.006	−0.313
*M. crispata*	16,592	33.6	36.7	18.6	11.1	70.3	−0.044	−0.25
*M. squinado*	16,598	33.7	37.1	18.2	11	70.8	−0.047	−0.245
*X. ngatama*	16,106	36.1	36.8	17.5	9.6	72.9	−0.01	−0.293
*A. lunaris*	15,807	34.8	35.4	18.7	11.1	70.2	−0.009	−0.256
*M. longicarpus*	15,548	32.4	36.6	19.2	11.8	69	−0.06	−0.236
*M. longipes*	16,424	37.5	34.2	18	10.4	71.7	0.046	−0.266
*C. bimaculata*	15,712	33.9	37.6	16.9	11.5	71.5	−0.052	−0.192
*T. thungwa*	16,156	37.2	36	9.1	17.6	73.2	0.017	0.318
*P. trituberculatus*	16,026	33.3	36.9	18.5	11.3	70.2	−0.051	−0.241
*C. sapidus*	16,263	34.2	34.9	19.8	11.1	69.1	−0.011	−0.279
*P. gigas*	15,515	35	35.5	18.7	10.8	70.5	−0.006	−0.268
*S. serrata*	15,775	34.6	38	17.1	10.4	72.6	−0.047	−0.242
*S. tranquebarica*	15,833	35	38.7	16.5	9.7	73.7	−0.05	−0.258
*S. olivacea*	15,723	33.5	35.9	19.4	11.2	69.4	−0.035	−0.267
*C. japonica*	15,738	33.8	35.4	18.9	11.9	69.2	−0.024	−0.228
*X. testudinatus*	15,798	36.7	37.2	16.8	9.3	73.9	−0.007	−0.286
*G. yunohana*	15,567	34.3	35.7	19.3	10.8	70	−0.019	−0.281
*A. distinguendus*	15,700	32.3	27.9	25.5	14.4	60.2	0.073	−0.278
*A. rodriguezensis*	15,611	35.3	33.5	20.9	10.3	68.8	0.025	−0.341
*P. crassipes*	15,652	30.5	35.8	21	12.7	66.3	−0.08	−0.245
*R. ranina*	15,557	30.3	36.3	21.1	12.2	66.6	−0.09	−0.266
*T. crenata*	15,787	34.4	35.3	18.8	11.5	69.7	−0.013	−0.24
*C. feriata*	15,660	34.1	36.1	18.6	11.3	70.2	−0.028	−0.246
*H. malayensis*	15,793	37.3	34.4	18.3	10	71.7	0.04	−0.292
*P. pelagicus*	16,157	33.7	35	19.1	12.2	68.7	−0.019	−0.219
*M. gladiator*	15,878	33.3	35.7	19.2	11.8	69	−0.034	−0.242
*N. minutum*	15,637	38	39.7	13.4	8.9	77.7	−0.022	−0.201
*C. haematocheir*	15,899	37.3	38.3	15	9.4	75.6	−0.013	−0.226
*E. anaglyptus*	16,435	33.2	34.8	21	11.1	68	−0.023	−0.309
*A. lactea*	15,659	34.8	34.6	18.5	12	69.4	0.003	−0.214
*P. subquadrata*	16,898	34.2	33.5	21.7	10.5	67.7	0.01	−0.347
*O. punctatus*	16,084	32.6	35.5	19.4	12.5	68.1	−0.042	−0.218
*L. depressum*	16,537	35.4	37.9	17.3	9.3	73.3	−0.034	−0.302

**Table 4:** Codon number and relative synonymous codon usage (RSCU) in *Grapsus albolineatus*. * represents the termination codon.

Codon	Count	RSCU	Codon	Count	RSCU	Codon	Count	RSCU	Codon	Count	RSCU
UUU (F)	233	1.43	UCU (S)	108	2.26	UAU (Y)	87	1.25	UGU (C)	25	1.61
UUC (F)	93	0.57	UCC (S)	37	0.77	UAC (Y)	52	0.75	UGC (C)	6	0.39
UUA (L)	229	2.42	UCA (S)	69	1.44	UAA (*)	8	1.6	UGA (W)	71	1.45
UUG (L)	80	0.85	UCG (S)	15	0.31	UAG (*)	2	0.4	UGG (W)	27	0.55
CUU (L)	102	1.08	CCU (P)	67	1.82	CAU (H)	45	1.08	CGU (R)	15	1.07
CUC (L)	36	0.38	CCC (P)	27	0.73	CAC (H)	38	0.92	CGC (R)	5	0.36
CUA (L)	96	1.01	CCA (P)	44	1.2	CAA (Q)	48	1.41	CGA (R)	29	2.07
CUG (L)	25	0.26	CCG (P)	9	0.24	CAG (Q)	20	0.59	CGG (R)	7	0.5
AUU (I)	201	1.32	ACU (T)	93	1.84	AAU (N)	76	1.2	AGU (S)	42	0.88
AUC (I)	103	0.68	ACC (T)	28	0.55	AAC (N)	51	0.8	AGC (S)	10	0.21
AUA (M)	163	1.5	ACA (T)	73	1.45	AAA (K)	67	1.51	AGA (S)	68	1.42
AUG (M)	54	0.5	ACG (T)	8	0.16	AAG (K)	22	0.49	AGG (S)	34	0.71
GUU (V)	107	1.57	GCU (A)	101	1.82	GAU (D)	44	1.19	GGU (G)	51	0.88
GUC (V)	17	0.25	GCC (A)	46	0.83	GAC (D)	30	0.81	GGC (G)	33	0.57
GUA (V)	116	1.7	GCA (A)	63	1.14	GAA (E)	53	1.36	GGA (G)	95	1.63
GUG (V)	33	0.48	GCG (A)	12	0.22	GAG (E)	25	0.64	GGG (G)	54	0.93

## Data Availability

The datasets generated for this study can be found in the GenBank accession no. MF198247.
